# Effectiveness of a comprehensive nursing prevention and control system for respiratory infections in fever clinics in the era of co-existence of multiple pathogens

**DOI:** 10.3389/fpubh.2026.1740733

**Published:** 2026-03-16

**Authors:** Hui Wang, Meijing Yan, Panpan Zhang, Jing Ma, Shanshan Zhao, Xiaoqing Li, Hong Dai, Yan Zhou, Ran Cui

**Affiliations:** 1 Beijing Youan Hospital, Capital Medical University, Infection General Fever Clinic, Beijing, China; 2 Beijing Youan Hospital, Capital Medical University, Information Network Management Center, Beijing, China

**Keywords:** co-existence of multiple pathogens, comprehensive nursing, fever clinic, prevention and control of nosocomial infection, respiratory tract infection

## Abstract

**Objective:**

To construct a comprehensive nursing prevention and control system for fever clinics that is suitable for an environment in which multiple pathogens coexist, to evaluate its effectiveness in preventing nosocomial cross-infection and to provide a preventable and controllable model that can be promoted in similar medical institutions.

**Methods:**

A retrospective cohort study was conducted involving 37,475 patients who attended the fever clinic of a tertiary hospital between January 2022 and March 2025. A total of 18,155 patients, treated between January 2022 and February 2023, were assigned to the control group, whereas 19,320 patients, treated between March 2023 and March 2025, were assigned to the observation group. The control group received routine nursing care, whereas the observation group received the comprehensive nursing prevention and control system proposed in this study, with “accurate identification, process optimisation, environmental and personnel protection and patient education” as its core components. Outcome measures included patient improvement before and after implementation of the comprehensive nursing prevention and control system, anxiety and depression scores, patient satisfaction and patients' mastery of educational content.

**Results:**

No cases of nosocomial infection were identified among patients, medical staff or accompanying persons before or after implementation of the comprehensive nursing prevention and control system. However, compared with the control group, the proportions of patients with multiple visits and those with aggravated conditions were both reduced in the observation group. In contrast, the number of patients with improved symptoms and the number of cases with consistent diagnoses increased significantly. Regarding scores on the Self-Rating Anxiety Scale (SAS) and the Self-Rating Depression Scale (SDS), the observation group had lower scores (SAS: 32.50 ± 8.32; SDS: 31.03 ± 10.77) than the control group (SAS: 39.20 ± 4.15; SDS: 42.60 ± 8.95; *P* < 0.005). With respect to patient satisfaction, the score in the observation group was 99.13 ± 1.46, compared with 91.13 ± 1.46 in the control group (*P* < 0.005). Regarding patients' mastery of the educational content, the total mastery rates in the control group and observation group were 11,699 (64.44%) and 17,638 (91.29%), respectively (*P* < 0.005).

**Conclusion:**

The comprehensive nursing prevention and control system constructed based on the reality of co-existence of multiple pathogens effectively prevents nosocomial cross-infection, significantly improves the nursing quality of fever clinics and patient satisfaction and provides a feasible and practical model and theoretical basis for infection prevention and control in medical institutions in the post-pandemic era.

## 1 Introduction

Prevention and control of hospital-acquired infections is an important component of medical quality management (1). It runs throughout the entire process of diagnosis and treatment. Effective measures for the prevention and control of hospital-acquired infections, together with standardized nursing management, play a crucial role in limiting the spread of infectious diseases (2). This importance is further amplified in the context of frequent public health emergencies (3). As the primary diagnostic sentinel for screening, preventing and controlling respiratory infectious diseases in medical institutions, fever clinics undertake the important function of early identification and isolation of patients at high risk and serve as a key barrier to blocking the in-hospital spread of pathogens (4).

The COVID-19 pandemic has profoundly reshaped the global landscape of respiratory infections. As China transitioned COVID-19 management to Category B, a classification denoting infectious diseases managed with routine prevention and control measures, the influenza virus, SARS-CoV-2, respiratory syncytial virus (RSV) and other pathogens have begun to co-circulate (5, 6). Surveillance data since 2023 have revealed overlapping seasonal peaks of these pathogens, leading to a more complex case mix in fever clinics. Symptom overlap among different infections complicates accurate diagnosis and may contribute to patient anxiety (7). Fever clinics now face three primary challenges (8): (1) pathogen diversity, requiring distinct control measures; (2) complexity of transmission routes, as pathogens can spread via droplets, contact and aerosols, increasing the risk of cross-infection in healthcare settings; and (3) high and variable patient flow, with large numbers of patients of all ages presenting during seasonal surges, heightening transmission risks.

In the face of this complex situation, pathogen-specific, stratified management has emerged as a key strategy (9–11). Accurate pathogen identification, risk assessment and tailored isolation can substantially reduce hospital-acquired infections (12). However, traditional models that focus on single pathogens (e.g. COVID-19 alone) are inadequate for this new era, as they fail to provide accurate triage for patients with overlapping symptoms. Furthermore, fever clinics often operate with limited staffing and space (13). There is an urgent need for a practical and comprehensive system that ensures effective multi-pathogen management and prevents in-hospital transmission under these constraints.

Therefore, this study develops and evaluates a comprehensive nursing prevention and control system for a tertiary hospital fever clinic. The system integrates four core modules: precise identification, process optimisation, environmental and personnel protection and patient education. By analyzing data from 37,475 patients, we assess its effectiveness in preventing nosocomial cross-infection to provide an evidence-based model for fever clinic management in the era of multiple co-circulating pathogens.

## 2 Materials and methods

### 2.1 General information

A convenience sampling method was used to select patients who attended the fever clinic of Beijing Youan Hospital, Capital Medical University, between January 2022 and March 2025 as participants in a retrospective cohort study to evaluate the implementation effect and clinical effectiveness of the comprehensive nursing prevention and control system. As the comprehensive nursing prevention and control system was implemented in our hospital in March 2023, 18,155 patients admitted between January 2022 and February 2023 were included in the control group, whereas 19,320 patients admitted between March 2023 and March 2025 were included in the observation group. This study included patients who completed diagnosis and treatment within the fever clinic setting and were subsequently discharged. Patients who were directly admitted to inpatient wards from the fever clinic because of severe conditions or specific diagnoses were excluded from the analysis in both groups. This study was approved by the Ethics Committee of Beijing Youan Hospital, Capital Medical University, and informed consent was obtained from both patients and their families.

The inclusion criteria were as follows: (1) patients attending the fever clinic with a primary diagnosis or chief complaint consistent with an acute respiratory tract infection (e.g. fever, cough, sore throat and dyspnoea); (2) age ≥18 years; (3) complete medical records; and (4) voluntary participation in the study with signed informed consent. The exclusion criteria were as follows: (1) fever ultimately attributed to non-respiratory causes (e.g. urinary tract infection or acute appendicitis) based on the final discharge diagnosis or definitive clinical or laboratory findings; (2) patients with malignant tumors; (3) patients with immunodeficiency; and (4) incomplete or missing key data in the hospital information system (e.g. missing diagnostic codes, incomplete visit records or absent follow-up information essential for outcome assessment).

Note: clarification on the application of exclusion criteria—Criterion 1 was applied retrospectively based on the final diagnosis established after completion of all clinically indicated investigations during the fever clinic visit or subsequent follow-up. This ensured that the study population was homogeneous with respect to acute respiratory tract infections. Criterion 4 was applied during data extraction and cleaning; patients whose electronic records were missing any data points required for the predefined outcome measures were excluded to maintain data integrity for analysis.

### 2.2 Method

#### 2.2.1 Members of the research group

Medical staff with more than 5 years of work experience in the fever clinic area were selected to form the research group, which consisted of three doctors and 15 nurses. Among these staff members, a head nurse was designated as the overall person in charge. All team members received unified training before commencement of the study and before implementation of the comprehensive nursing intervention for the observation group. The training covered the knowledge base, methodologies, precautions and psychological nursing components of the comprehensive nursing prevention and control system.

#### 2.2.2 Control group

Patients received routine nursing care in accordance with the hospital's standard operating procedures for fever clinics at that time, which were not structured under the comprehensive nursing prevention and control system. The specific practices were as follows:

Triage and initial assessment: Upon arrival, patients underwent simple triage conducted by a triage nurse. This primarily involved recording basic information, enquiring about core symptoms (fever, cough and sore throat) and measuring body temperature. Based on this initial screening, patients were directed to the general waiting area.

Diagnostic approach: Diagnostic testing was not routinely performed at the point of triage for all patients. Testing for influenza and SARS-CoV-2 (typically via antigen tests) was ordered at the discretion of the attending physician based on clinical presentation during the consultation, rather than as a standardized pre-consultation screening tool.

Infection prevention and control measures:

Patient flow and isolation: There was no dedicated or separate channel exclusively for patients with fever; they often shared common routes with other outpatients.

Personal protective equipment (PPE): Medical staff followed standard droplet and contact precautions, which typically included wearing surgical masks, gloves and gowns when in close contact with patients. However, there were no formal or periodic inspections of PPE donning and doffing or mask fit.

Patient education and discharge: After diagnosis, patients received simple, non-standardized oral instructions on home care and general precautions (e.g. rest, hydration and fever management).

#### 2.2.3 Observation group

A comprehensive nursing prevention and control system centered on “accurate identification, process optimisation, environmental and personnel protection and patient education” was adopted for management. The specific nursing operations of each module were as follows:

**Part 1, Accurate Identification**: Nurses served as the main body responsible for initial assessment and identification, adopting a two-dimensional triage process comprising initial symptom screening and rapid testing.

Initial symptom screening: Through standardized enquiry and observation, patients were divided into two categories—(1) patients with combined symptoms of fever and cough (body temperature ≥37.3 °C accompanied by dry cough or expectoration) and (2) patients with a symptom of cough (no fever or body temperature <37.3 °C) or fever alone (no cough, body temperature ≥37.3 °C). Patients in the combined symptom group were preferentially classified as “high risk for examination”, whereas those in the single cough or fever group were classified as “routine monitoring”. The two patient categories wore red and blue identification wristbands, respectively, to enable visual differentiation.

Rapid testing: Nurses assisted with sample collection for influenza nucleic acid or antigen testing and SARS-CoV-2 nucleic acid or antigen testing. During the study period, antigen testing was the most commonly used rapid detection method because of its short turnaround time, typically 15–30 min, lower cost and ease of administration in an outpatient setting, which facilitated timely triage and initial diagnosis. Further triage was conducted based on test results: patients with positive results were guided by nurses to the specialized infectious disease clinic, whereas patients with negative results were assigned to the general fever clinic or general respiratory clinic according to symptom type, such as fever, productive cough or dyspnoea. The entire triage process was completed within 30 min to minimize cross-contact between patients with different risk profiles.

**Part 2, Process Optimisation**: A nursing support process centered on independent channel guidance and priority consultation was designed to reduce infection risk during the waiting period.

Independent channel guidance: Nurses established dual-route guiding signage at the entrances on the first and second floors of the fever clinic to clearly mark the patients with fever channel. One full-time nurse was assigned to guide patients to avoid the intersection of patient routes. For older patients or children with limited mobility, nurses provided wheelchairs or pushcarts to assist them in entering the waiting area via the independent channel.

Control of waiting area congestion: Time-slot-based waiting was implemented through the hospital information system to ensure that the number of patients waiting in each time slot did not exceed 20. Waiting reminder text messages were sent via the information system, including the current number of waiting patients and the estimated consultation time. Seating guidance signs were placed on every other seat in the waiting area. Nurses conducted regular patrols of the waiting area and guided patients to sit at appropriate intervals to prevent crowding and close contact.

**Part 3, Environmental and Personnel Protection**: Centered on maintaining a sterile environmental and standardized protection, nurses fulfilled defined responsibilities in the execution and guidance of protective measures.

Environmental protection nursing: Nurses strictly implemented comprehensive disinfection at least five times per day, specifically after three meals and after peak periods of patient attendance. Disinfection focused on high-frequency contact surfaces such as examination desks, doorknobs, stethoscopes and sphygmomanometers, which were wiped with 75% alcohol with a contact time of at least 3 min. Consulting room windows were opened once per hour for 15 min to ensure adequate daily ventilation. In the outpatient department, a fresh air system was used to maintain appropriate air circulation.

Personnel protection guidance and supervision: (1) Protection of medical staff—before commencing work, medical staff were required to pass inspections related to donning and doffing of protective equipment and disinfection procedures, including correct use of isolation gowns, hats and gloves; verification of mask fit; and performance of hand disinfection before and after procedures. Staff were supervised to strictly adhere to principles of aseptic operation. During working hours, the nursing team leader inspected the use of protective equipment every 4 h. If any looseness or damage was identified, immediate assistance with replacement was provided. (2) Protection of patients and accompanying persons—when patients entered the fever clinic, masks and instruction cards on correct mask use were distributed by the hospital, and nurses demonstrated the correct wearing method on site. During waiting and consultation periods, patrols were conducted every 20 min. Patients or accompanying persons wearing masks incorrectly, such as exposing the nose or mouth or having loose ear loops, were promptly corrected to ensure full compliance with protection requirements.

**Part 4, Patient Education**: Centered on three core elements, namely respiratory hygiene, home isolation and indications for follow-up visits, a nurse-led hierarchical education process was implemented.

Respiratory hygiene guidance: Nurses instructed patients and their families in cough etiquette, including covering the mouth and nose with the inner elbow rather than the hands when coughing or sneezing and promptly disinfecting hands with hand sanitizer afterwards. Education was delivered through oral explanation and practical demonstration, and the illustrated *Respiratory Hygiene Handbook* was distributed.

Home isolation advice: For patients with positive test results or those assessed as high risk, nurses developed a home isolation care checklist that clearly stated four core requirements—(1) live alone, or if an independent room was unavailable, maintain a distance of at least 2 m from family members; (2) wipe household surfaces twice daily using a chlorine-containing disinfectant at a concentration of 500 mg/L; (3) use separate tableware, with tableware boiled for 15 min after meals; and (4) avoid going out. If going out was unavoidable, patients were instructed to wear an N95 mask.

Notification of indications for follow-up visits: Nurses clearly informed patients, through key point highlighting and oral emphasis, that an immediate follow-up visit was required if one or more of the following five conditions occurred—(1) persistent fever lasting more than 3 days with a body temperature ≥39 °C; (2) dyspnoea or chest pain; (3) confusion or lethargy; (4) worsening cough accompanied by haemoptysis; and (5) difficulty eating or reduced urine output.

**Part 5, Psychological Support**: Nurses proactively attended to patients' emotional states during routine nursing care and health education. Specific measures included the following.

Pathogen knowledge education: Plain language was used to explain the characteristics of co-existing multiple pathogens, their main transmission routes and the effectiveness of the hospital's prevention and control measures to alleviate patients' fear of unknown infections and disease recurrence.

Relaxation skills guidance: For patients with evident anxiety, such as restlessness or frequent enquiries, nurses guided them to practice simple relaxation techniques, including slow deep breathing (inhaling for 4 s, holding for 2 s and exhaling for 6 s) and progressive muscle relaxation, in which muscle groups from the head to the feet were alternately tensed and relaxed for 5–10 min per session.

Emotional communication channel: Dedicated time, approximately 5–10 min per patient, was allocated to listen to patients' concerns and questions. Nurses responded positively and patiently and avoided perfunctory replies. Patients with severe negative emotions were promptly referred to the hospital's psychological counseling team for further intervention.

### 2.3 Evaluation method

#### 2.3.1 Indicators of general improvement in patients

Patients with multiple visits refer to patients who repeatedly attended the fever clinic for the same respiratory infection during the study period. Patients with improved symptoms refer to patients whose core symptoms, such as fever and cough, were considerably alleviated after the intervention (e.g. body temperature returned to normal and cough frequency decreased by ≥50 %). Consistent diagnosis refers to patients whose diagnostic results remained unchanged between the initial diagnosis and subsequent diagnostic and treatment processes. Patients with aggravated conditions refer to patients who experienced symptom deterioration, such as persistent high fever or new-onset dyspnoea, or developed complications after the intervention. Nosocomial infection refers to infections acquired by patients within the hospital, including infections that occurred during the hospital stay and those that occurred after discharge but were acquired in the hospital. This definition excludes infections that began before hospital admission or were present at the time of admission. Infections acquired by medical staff and accompanying persons within the hospital were also classified as nosocomial infections. This indicator was determined based on a combination of symptom changes, laboratory test results and contact history.

#### 2.3.2 Anxiety and depression scales

The nursing team assigned a trained research nurse to guide participants in completing evaluations of anxiety and depression levels before the intervention, either before or during the medical visit and 1 week after the intervention through online completion. The research nurse also compiled and analyzed the results of the two assessments. The Self-Rating Anxiety Scale (SAS) and the Self-Rating Depression Scale (SDS) were developed by Zung in 1971 and 1965, respectively (14). Each scale comprises 20 items. An SAS score of ≥50 indicates the presence of anxiety, with higher scores reflecting greater severity (Cronbach's α = 0.91). An SDS score of ≥53 indicates the presence of depression, with higher scores reflecting greater severity (Cronbach's α = 0.88).

#### 2.3.3 Patient satisfaction survey

Research nurses organized patients to scan the QR code for the satisfaction survey of Beijing Youan Hospital, Capital Medical University, and complete the questionnaire at the end of their visits. The questionnaire collected information on patients' names and departments and assessed aspects such as the environment in the isolation area, technical proficiency and service quality. It consisted of 10 items with five response options ranging from “Very satisfied” to “Very dissatisfied”, scored as 10, 8, 6, 4 and 2 points, respectively, for a total possible score of 100. Higher scores indicated higher levels of patient satisfaction.

#### 2.3.4 Patients' mastery of health knowledge survey

A questionnaire assessing patients' mastery of health knowledge, developed by the hospital, was used. A QR code was generated using Wenjuanxing software (Changsha Ranxing Info-Tech, Changsha, China). Upon leaving the hospital, patients were guided by nurses to scan the code and complete the questionnaire. The questionnaire covered three domains, namely respiratory hygiene guidance, home care advice and notification of follow-up indications, and it consisted of a total of 10 items. Each item was scored from 0 to 10 points based on answer accuracy, yielding a total possible score of 100 points. Patients were categorized into three groups according to their scores: fully mastered (>90 points), partially mastered (60–90 points) and not mastered (<60 points). Before formal use of the questionnaire, reliability and validity testing was conducted. Content validity was evaluated using expert assessment, with a content validity index of 0.92, indicating good relevance and comprehensiveness. Internal consistency was assessed using Cronbach's α coefficient, which was 0.87, demonstrating good internal consistency. Test–retest reliability was evaluated using the intraclass correlation coefficient (ICC). Thirty patients completed the questionnaire twice at a 2-week interval, yielding an ICC of 0.85, indicating good stability. The calculation formula for the mastery rate of health education content was as follows: overall mastery rate = (number of cases with full mastery + number of cases with partial mastery)/total number of cases × 100%.

### 2.4 Data collection

During the study period, research nurses regularly collected and organized patients' basic information (e.g. age, gender, diagnosis and medication) and indicators of clinical improvement (e.g. number of repeat visits, number of patients with improved or aggravated conditions and number of patients with nosocomial infections) every week using the hospital information management system. Assessment of psychological status, patient satisfaction and mastery of educational content was primarily conducted using the SAS and SDS scales and online questionnaires, with patients reminded to complete them upon leaving the hospital. Research nurses reviewed completed scales and questionnaires weekly. For incomplete forms, completion was facilitated through system prompts to ensure data completeness.

### 2.5 Sampling method and consideration of potential bias

This study employed a convenience sampling method to select patients from the fever clinic. Although this approach is pragmatic for retrospective cohort studies conducted in busy clinical settings and facilitates the inclusion of a large sample over a defined period, it carries a risk of selection bias. The patients included may not be fully representative of all individuals presenting to the fever clinic during the study period. To minimize the potential impact of bias on cohort comparisons and to strengthen the internal validity of the analysis, the following measures were implemented.

First, strict, pre-defined inclusion and exclusion criteria were applied uniformly to both the control and observation groups. This ensured that the cohorts were constructed using the same clinical parameters, such as age ≥18 years, a primary complaint of fever or respiratory symptoms and completion of care within the fever clinic. Second, to reduce information bias and ensure data completeness, all patient data were extracted from the structured hospital information management system. These data included demographic characteristics, visit records, diagnostic codes and test results, thereby reducing reliance on potentially incomplete manual documentation. Third, baseline characteristics, including age, gender and diagnostic distribution, were formally compared between the control and observation groups. The absence of statistically significant differences in these key variables supports the comparability of the two cohorts for evaluation of the intervention effect despite the use of convenience sampling. Finally, the exclusion of patients directly admitted from the fever clinic was explicitly stated, clarifying the study population and avoiding misinterpretation of outcomes in patients with more severe conditions.

### 2.6 Statistical methods

For data analysis, SPSS version 26.0 was used. Continuous variables with a normal distribution were expressed as mean ± standard deviation, and comparisons between groups were performed using the *t*-test. Categorical variables were expressed as percentages and analyzed using the χ^2^ test. A *P*-value <0.05 was considered statistically significant.

## 3 Results

### 3.1 Basic information of the patients

Before the main outcome analyses were conducted, baseline characteristics of patients in the two groups were compared to assess the potential influence of confounding factors on the results. Comparisons included age distribution, gender and diagnostic distribution. Statistical analysis showed no significant differences between the control group and the observation group in age distribution, gender or diagnostic distribution (*P* > 0.05), indicating good comparability between the groups (see Table 1).

**Table 1:** Patient basic information [*n* (%)].

**Project**	**Sample situation (*n* = 37,475)**	**Observation group (*n* = 19,320)**	**Control group (*n* = 18,155)**	***t***	***P***
**Age**				1.668	0.096
Adult (18–64)	29,675 (79.18)	15,300 (79.19)	14,375 (79.18)		
Elder (≥65)	4,204 (11.22)	2,200 (11.39)	2,004 (11.04)		
Children (<18)	3,596 (9.6)	1,820 (9.42)	1,776 (9.78)		
**Gender distribution**				1.328	0.249
Male	18,428 (49.18)	9,526 (49.31)	8,902 (49.03)		
Female	19,047 (50.82)	9,794 (50.69)	9,253 (50.97)		
**Diagnostic distribution**				2.645	0.196
Fever symptoms	22,162 (59.14)	11,581 (59.94)	10,581 (58.28)		
Influenza A	6,764 (18.05)	3,600 (18.63)	3,164 (17.43)		
Influenza B	1,335 (3.56)	691 (3.58)	644 (3.55)		
Common cold	1,064 (2.84)	563 (2.91)	501 (2.76)		
COVID-19	171 (0.47)	89 (0.46)	82 (0.45)		
Others	5,979 (15.95)	3,102 (16.06)	2,877 (15.85)		

### 3.2 Improvement of patients' conditions

Compared with the control group, the observation group showed significantly lower proportions of multiple visits and aggravated conditions and significantly higher proportions of symptom improvement and consistent diagnoses. No nosocomial infections occurred in either group (see Table 2).

**Table 2:** Evaluation of the implementation effect of the comprehensive prevention and control system [*n* (%)].

**Evaluation index**	**Observation group (*n* = 19,320)**	**Control group (*n* = 18,155)**	***P***
Patients who have visited the hospital multiple times	9,095 (47.8)	12,115 (66.73)	<0.001
Patients with improved symptoms	2,102 (10.88)	1,533 (8.45)	<0.001
The diagnosis remains consistent	8,801 (45.55)	6,987 (38.49)	<0.001
Patients with aggravated conditions	83 (0.43)	310 (1.71)	<0.001
Nosocomial infection	0	0	<0.001

### 3.3 Comparison of psychological state, satisfaction and mastery of educational content

The observation group demonstrated significantly lower SAS and SDS scores, significantly higher satisfaction scores and a significantly higher overall mastery rate of educational content than the control group (all *P* < 0.05; see Table 3).

**Table 3:** Comparison of psychological state, satisfaction, and mastery of educational content [*x* ± *s, n* (%)].

**Indicator**		**Observation group (*n* = 19,320)**	**Control group (*n* = 18,155)**	***t***	***P***
SAS	Before intervention	62.30 ± 3.15	61.10 ± 2.27	0.256	0.623
	After intervention	32.50 ± 8.32	39.20 ± 4.15	2.697	0.015
	*t*	9.577	4.531		
	*P*	<0.001	<0.001		
SDS	Before intervention	64.60 ± 10.16	63.43 ± 7.63	1.579	0.163
	After intervention	31.03 ± 10.77	42.60 ± 8.95	2.109	0.010
	*t*	13.625	11.634		
	*P*	<0.001	<0.001		
Satisfaction score		99.13 ± 1.46	91.13 ± 1.46	2.431	0.018
Mastery of educational content	Not mastered	1,682 (8.71)	6,456 (35.56)		
	Partial mastery	5,210 (26.97)	8,584 (47.28)		
	Master completely	12,428 (64.33)	3,115 (17.16)		
	Overall mastery degree	17,638 (91.29)	11,699 (64.44)	50.281	<0.001

### 3.4 Seasonal distribution of patient admissions

**Figure 1:**
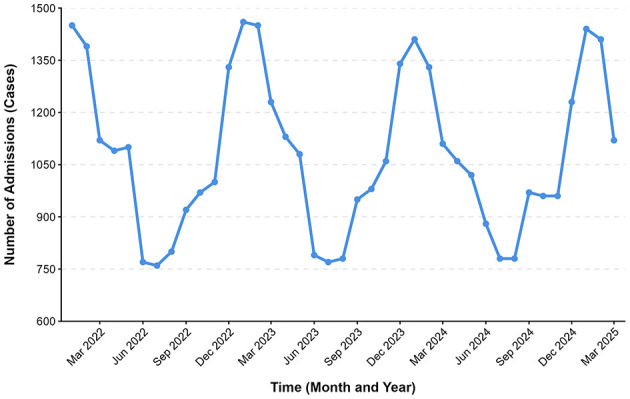
Seasonal distribution of monthly admissions to the fever clinic (January 2022–March 2025).

Between January 2022 and March 2025, the number of fever clinic admissions exhibited clear seasonal fluctuations (see Figure 1). Winter (December–February) had the highest monthly admission volume, with an average of 1,428 cases per month, followed by spring (March–May), with an average of 1,105 cases per month. Summer (June–August) had the lowest admission volume, averaging 783 cases per month, whereas autumn (September–November) showed a gradual upward trend, with an average of 967 cases per month.

## 4 Discussion

In the post-pandemic period, influenza, COVID-19, RSV and other pathogens have co-circulated, creating a co-epidemic landscape. As the frontline of infection control, fever clinics must address pathogen diversity, complex transmission routes and high patient volumes (15–17). The four-module nursing system evaluated in this study effectively addressed these challenges. Notably, it resulted in zero cases of nosocomial cross-infection among patients, medical staff or accompanying persons. In addition, several patient outcomes significantly improved: the symptom improvement rate increased to 10.88% compared with 8.45% in the control group, diagnostic consistency reached 45.55% compared with 38.49% and the condition deterioration rate decreased to 0.43% compared with 1.71%. These findings support the effectiveness and comprehensiveness of the proposed model.

In this study, nurses served as the primary personnel for initial assessment and identification, achieving accurate triage within 30 min through symptom stratification and rapid testing. This design is highly consistent with the findings reported by Qu (18). That study implemented a three-level pre-examination and triage process and strictly enforced a system of three areas and two channels, namely dedicated channels for medical staff and for patients with fever, thereby achieving zero in-hospital cross-infection. In addition, the use of rapid testing in the present study aligns with the practical trend toward precision medicine in infection prevention and control. Peng et al. (12) reported that fever clinics could timely and accurately triage patients at high risk through three-level pre-examination and triage, thereby reducing unnecessary personnel flow and the risk of nosocomial infection. This finding is consistent with the zero cross-infection observed in the present study and indicates that accurate identification is a critical first step in interrupting transmission chains involving multiple pathogens.

The process design incorporating independent channels and priority consultation represents another key factor in reducing infection risk in this study. Previous research has shown that in densely populated settings, non-pharmaceutical interventions, such as mask wearing and maintenance of social distancing, can effectively reduce the spread of respiratory infections (19–21). The present study applied these principles in two ways. First, dual flow-line separation of patients with fever and general patients was implemented, directly reducing the intersection between different patient groups and effectively limiting droplet and contact transmission through physical spatial separation (22). Second, a staggered appointment and waiting strategy was adopted. In addition, on-site patrols were conducted to guide patients to sit at appropriate intervals and to wear masks correctly, further reducing the risk of clustered transmission. According to the results, the proportion of patients with multiple consultations in the observation group (47.08%) was significantly lower than that in the control group (66.73%). This finding indirectly suggests that process optimisation not only reduced infection risk but also decreased the likelihood of repeated consultations due to recurrent conditions by shortening waiting times and improving diagnostic and treatment efficiency.

The graded education on respiratory hygiene, home isolation and indications for follow-up visits represents an extended component of the prevention and control system evaluated in this study. Its core value lies in extending prevention and control efforts from the hospital to the home setting. The results showed that overall mastery of educational content among patients in the observation group was higher than that in the control group, indicating that systematic patient education can improve self-protection compliance among patients with respiratory infections. Specifically, this improvement was reflected in increased rates of correct mask use and appropriate home disinfection practices, thereby reducing intra-household transmission and revisit rates, which is consistent with the findings reported by Chen (23). Although this study did not directly measure intra-family transmission rates or establish a causal link between specific home care activities and psychological state, the significant improvement in patients' mastery of health knowledge, coupled with the observed reductions in anxiety and depression scores, suggests that empowering patients with clear and actionable home care instructions may enhance their sense of control and safety. Such perceived control is a recognized factor in mitigating pandemic-related anxiety and depression. The potential reduction in intra-family transmission, inferred from improved hygiene practices, warrants further targeted investigation.

In addition, nurses in this study clearly identified five categories of indications for follow-up visits through key point marking and oral emphasis, which were directly associated with a reduction in disease deterioration rates. Previous studies have indicated that clear and systematic follow-up guidance can reduce the proportion of patients whose conditions worsen because of delayed treatment (24, 25). This is supported by the low disease deterioration rate of 0.43% observed in the observation group in the present study, indicating that patient education is not merely a supplementary component of prevention and control but a critical factor in improving patient prognosis.

Although this study focused on infection prevention and control, it also included the assessment of anxiety and depression scores. After the intervention, both anxiety and depression scores in the observation group were significantly lower than those in the control group, and the differences within each group before and after the intervention were statistically significant (*P* < 0.001). This finding is not incidental. Previous studies have shown that in closed environments where multiple pathogens coexist, fear of infection with unknown pathogens and concerns about disease recurrence can significantly increase the incidence of anxiety and depression among patients with fever, and such negative emotions may delay symptom recovery by affecting immune function (26). In this study, psychological support was incorporated into the comprehensive nursing system as an independent module, including pathogen knowledge popularization, relaxation skill guidance and emotional communication channels. These specific measures helped to address patients' psychological concerns at the source and provided actionable emotional support strategies, which is an important reason for the significant reduction in anxiety and depression scores in the observation group. It should be noted, however, that the increased attention and structured interaction from nurses in the observation group, through activities such as teaching, movement guidance, health education and frequent environmental disinfection, may have introduced a Hawthorne effect. This effect, whereby participants modify their behavior or reporting simply because they are aware that they are being studied or receiving additional attention, could partially account for the improvements observed in self-reported outcomes such as anxiety, depression and satisfaction. Enhanced monitoring and support may also have positively influenced patients' perceptions of care and their engagement in the recovery process, thereby contributing to greater diagnostic stability and symptom improvement. Although the core infection control measures (e.g. strict disinfection, PPE use and patient flow management) have a direct mechanistic basis for preventing nosocomial transmission, as evidenced by the zero infection result, the psychological and satisfaction outcomes should be interpreted with this potential bias in mind. This further indicates that the comprehensive nursing system not only focuses on infection prevention and control but also considers patients' overall health, reflecting the humanistic nature of modern nursing.

Patient satisfaction refers to patients' perceptions of the extent to which their needs or expectations for healthcare services are fulfilled (27). The technical level, work status, service attitude, health education and humanistic care provided by medical service personnel directly affect patient satisfaction (28). In this study, patient satisfaction in the observation group was higher than that in the control group (*P* < 0.05), which is closely related to the symptom pre-screening and rapid testing strategy, as well as the hierarchical education on respiratory hygiene, home isolation and follow-up indications. Rapid triage and consultation, timely identification of the cause of illness and clear, systematic follow-up guidance all contribute to improving patients' consultation experience.

The seasonal distribution of admissions, with a winter peak and summer trough, is consistent with the epidemiological characteristics of respiratory pathogens such as influenza and RSV (5, 7). This finding suggests that fever clinics should adjust nursing resources according to seasonal variations, for example, by increasing staffing levels and optimizing triage processes during winter peaks to improve service efficiency.

Limitations: First, this study adopted a single-center retrospective design with a convenience sampling method, based on data from a tertiary grade A hospital. As a result, selection bias may be present, and the findings should be interpreted with caution when generalizing to primary hospitals or other regions. Second, the study did not track long-term indicators such as reinfection rates or home isolation compliance 3–6 months after discharge, making it impossible to fully evaluate the indirect effects of the system on community-level prevention and control. Third, the potential impact of specific home care education components on psychological outcomes and intra-family transmission was not quantitatively assessed, which limits the ability to directly attribute the observed benefits to these activities. Fourth, as acknowledged above, the study design could not fully exclude the potential influence of the Hawthorne effect on patient-reported outcomes (e.g. anxiety, depression and satisfaction) and possibly on clinician behavior, affecting diagnostic consistency. Future studies incorporating blinded outcome assessment or alternative control designs would help to mitigate this concern. Finally, no subgroup analysis was conducted to examine the prevention and control effects for different pathogens, such as influenza A, COVID-19 and influenza B, which limits conclusions regarding the system's adaptability to specific pathogens.

In future research, multi-center prospective studies should be conducted, incorporating samples from primary hospitals and different regions, to further verify the generalizability of the system. In addition, module details could be optimized for different pathogens, for example, by adjusting rapid testing items and refining disinfection frequencies, to enhance the pathogen-specific adaptability of the system.

## 5 Conclusion

The comprehensive nursing prevention and control system developed in this study was associated with the prevention of nosocomial cross-infection, as well as improvements in the nursing quality of fever clinics and patient satisfaction. It also appeared to alleviate patients' anxiety and depression to some extent, which may be conducive to disease recovery. Accordingly, this system provides a preliminary practical model and theoretical basis for infection prevention and control in medical institutions in the post-pandemic era. It is worth emphasizing that the core components of this system—meticulous triage, strict environmental and personnel infection control and structured patient education—represent foundational, high-standard care that is arguably essential in fever clinic settings managing multiple transmissible pathogens. The observation of zero nosocomial infections alongside improved patient outcomes supports the view that such comprehensive nursing care should not be regarded merely as an experimental intervention but should be integrated into the routine standard of care for fever clinics operating in similarly complex epidemiological environments. The key challenge lies not in demonstrating benefit, but in achieving sustainable implementation under resource constraints. Nevertheless, these findings should be interpreted in light of the study's limitations, including the use of convenience sampling in a single-center retrospective design and the potential influence of unmeasured temporal confounding factors, such as changes in community pathogen prevalence over time. In addition, this study offers an operable practical model for fever clinics in the context of co-circulating multiple pathogens and may serve as a reference for infection prevention and control policies in medical institutions. On the one hand, the nurse-led and standardized operational design of the system could be considered for incorporation into the implementation guidelines of the Standard for Standardized Construction of Fever Clinics to inform nursing practice at different levels of hospitals. On the other hand, the achievement of zero nosocomial cross-infection and high patient satisfaction provides a potential benchmark for management goals that balance infection prevention and control with patient experience in the post-pandemic era. Future research is needed to promote the adoption of this comprehensive nursing model across a wider range of medical institutions, to continuously refine it through large-scale implementation and to explore its potential as a Chinese solution for nursing prevention and control in fever clinics under conditions of multiple pathogen co-circulation.

## Data Availability

The original contributions presented in the study are included in the article/supplementary material, further inquiries can be directed to the corresponding author.
